# GMSC-Derived Exosomes Combined with a Chitosan/Silk Hydrogel Sponge Accelerates Wound Healing in a Diabetic Rat Skin Defect Model

**DOI:** 10.3389/fphys.2017.00904

**Published:** 2017-11-07

**Authors:** Quan Shi, Zhiyong Qian, Donghua Liu, Jie Sun, Xing Wang, Hongchen Liu, Juan Xu, Ximin Guo

**Affiliations:** ^1^Department of Stomatology, Chinese PLA General Hospital, Beijing, China; ^2^School of Biological Science and Medical Engineering, Beihang University, Beijing, China; ^3^Department of Advanced Interdisciplinary Studies, Institute of Basic Medical Sciences and Tissue Engineering Research Center, Academy of Military Medical Sciences, Beijing, China; ^4^Stomatology Center, General Hospital of Armed Police Forces, Beijing, China

**Keywords:** gingival mesenchymal stem cells, exosomes, hydrogel sponge, diabetic rats, skin repair and regeneration

## Abstract

**Background:** Delayed wound healing in diabetic patients is one of the most challenging complications in clinical medicine, as it poses a greater risk of gangrene, amputation and even death. Therefore, a novel method to promote diabetic wound healing is of considerable interest at present. Previous studies showed that injection of MSC-derived exosomes has beneficial effects on wound healing. In current studies, we aimed to isolate exosomes derived from gingival mesenchymal stem cells (GMSCs) and then loading them to the chitosan/silk hydrogel sponge to evaluate the effects of this novel non-invasive method on skin defects in diabetic rats.

**Methods:** GMSCs were isolated from human gingival connective tissue and characterized by surface antigen analysis and *in vitro* multipotent differentiation. The cell supernatant was collected to isolate the exosomes. The exosomes were characterized by transmission electron microscopy, Western blot and size distribution analysis. The chitosan/silk-based hydrogel sponge was prepared using the freeze-drying method and then structural and physical properties were characterized. Then, the exosomes were added to the hydrogel and tested in a diabetic rat skin defect model. The effects were evaluated by wound area measurement, histological, immunohistochemical and immunofluorescence analysis.

**Results:** We have successfully isolated GMSCs and exosomes with a mean diameter of 127 nm. The chitosan/silk hydrogel had the appropriate properties of swelling and moisture retention capacity. The *in vivo* studies showed that the incorporating of GMSC-derived exosomes to hydrogel could effectively promote healing of diabetic skin defects. The histological analysis revealed more neo-epithelium and collagen in the hydrogel-exosome group. In addition, the hydrogel-exosome group had the highest microvessel density and nerve density.

**Conclusions:** The combination of GMSC-derived exosomes and hydrogel could effectively promote skin wound healing in diabetic rats by promoting the re-epithelialization, deposition and remodeling of collagen and by enhancing angiogenesis and neuronal ingrowth. These findings not only provide new information on the role of the GMSC-derived exosomes in wound healing but also provide a novel non-invasive application method of exosomes with practical value for skin repair.

## Introduction

As an important cause of death and disability worldwide, diabetes mellitus (DM) is characterized by high blood sugar levels over a prolonged period. There are 422 million DM patients registered globally (2014) according to the WHO, and this number is expected to rise to 592 million by 2035 (Ledford, 2013; Krug, 2016). Many factors, including high glucose burden, impaired angiogenesis, and imbalance in cytokine profile, contribute to the chronic, delayed and even non-healing wounds in DM patients (Franzén and Roberg, 1995; Lerman et al., 2003; Berlangaacosta et al., 2012; Xuan et al., 2014). Delayed wound healing is one of the most challenging complications in clinical medicine, as it poses a greater risk of gangrene, amputation, and even death (Greenhalgh, 2003). More importantly, delayed wound healing not only leads to increasing healthcare costs but also seriously impairs the quality of life in DM patients. Because the traditional therapies are often insufficient, a novel method to promote diabetic wound healing is of considerable interest at present (Hyldig et al., 2017).

Exosomes are small membrane vesicles of endocytic origin that are secreted by most cells. As a naturally secreted nanoparticle ranging in size from 30 to 150 nm, exosomes are widely involved in cell-to-cell communication, which plays important roles in tissue repair and regeneration, disease diagnosis and oncobiology (Kourembanas, 2015; Rani and Ritter, 2016; Tkach and Thery, 2016). Exosomes represent an important mode of intercellular communication by serving as vehicles for the transfer of membrane and cytosolic proteins, lipids and RNAs between cells (Raposo and Stoorvogel, 2013). Recent studies have shown that many stem cell-derived exosomes can enhance wound healing and facilitate skin regeneration, as well as diabetic skin wound healing, by promoting proliferation and migration of related cells, enhancing angiogenesis, re-epithelization, and regulating immune responses. Together, these results revealed that the use of exosomes may be a promising approach to achieve a cell-free alternative to stem cell therapy (Kourembanas, 2015; Zhang et al., 2015a,c; Li et al., 2016; Phinney and Pittenger, 2017).

Gingival mesenchymal stem cells (GMSCs) are a group of stem or progenitor cells isolated from the gingival lamina propria (Zhang et al., 2012; El-Sayed et al., 2015; Jin et al., 2015). Compared with other mesenchymal stem or progenitor cell sources, GMSCs are abundant and easily obtainable (Fawzy El-Sayed and Dörfer, 2016). Furthermore, GMSCs have stronger proliferation ability and more stable morphology than bone marrow MSCs. More importantly, GMSCs show remarkable tissue regenerative potential and noteworthy immunomodulatory properties (Tomar et al., 2010; Zhang et al., 2012; Fawzy El-Sayed and Dörfer, 2016). Previous studies have shown that the GMSCs can enhance wound healing (Zhang et al., 2010; Jiang et al., 2015), therefore, we hypothesized that GMSC-derived exosomes may also promote tissue repair during wound healing. However, the GMSC-derived exosomes have not yet been isolated and characterized.

Biomaterial-based wound dressings, which have been receiving considerable research interest, also play a critical role in wound healing and skin regeneration. As a cationic natural polymer, chitosan has been widely used for wound dressing and as a drug-delivery vehicle to improve wound healing because of its hemostatic, anti-microbial, biocompatible and biodegradable properties (Dai et al., 2011). In addition, the silk-based hydrogel has also been shown to have beneficial effects on wound healing; the beneficial effects were not only because silk can promote cell attachment, proliferation and extracellular matrix (ECM) production but also silk can enhance the mechanical strength of the hydrogel obtained from other natural polymers (Kapoor and Kundu, 2016). Hence, combining chitosan with silk is a good option for suitable wound dressing to promote the skin repair and regeneration (Kapoor and Kundu, 2016). At present, exosomes are mainly applied through subcutaneous injection to several sites around the wound, and only a few studies used exosome scaffolds as wound dressing for skin wound healing. To make the exosomes more realistic in clinical application, a simple, effective and non-invasive method is needed. Therefore, a porous chitosan/silk hydrogel sponge may be one of the choices to achieve the delivery of the exosomes.

**Figure 1:**
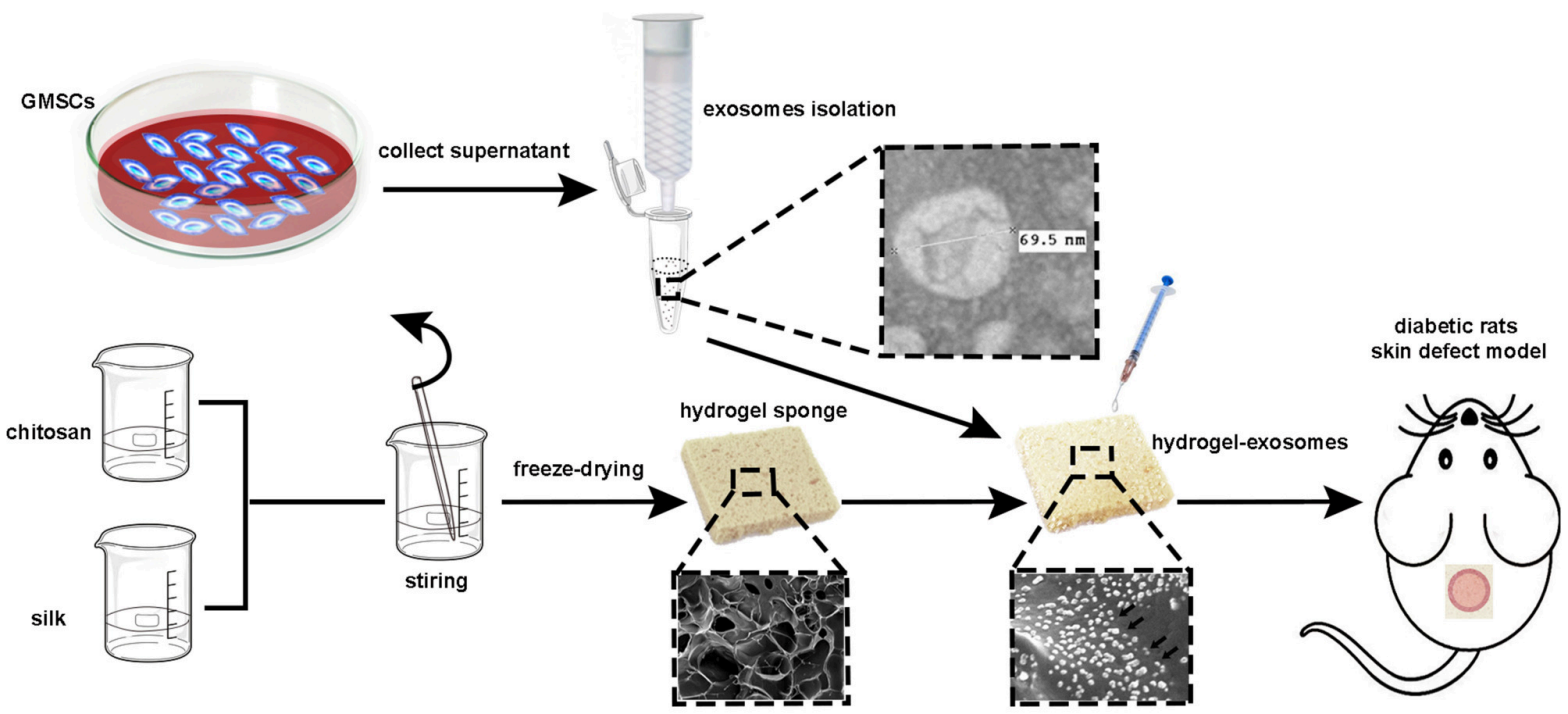
Schematic illustration of the isolation of GMSC-derived exosomes and preparation of chitosan/silk hydrogel for a diabetic rat skin defect model.

In the current study, our aim is to isolate exosomes derived from GMSCs, loading the GMSC-derived exosomes to the hydrogel sponge to achieve efficient delivery of GMSC-derived exosomes directly to skin wounds and then evaluate the healing effects in a streptozotocin (STZ)-induced diabetic rat skin defect model (the schematic illustration is shown in Figure 1).

## Materials and methods

### Isolation of human gingival mesenchymal stem cells

All the obtained human tissue specimens and cell isolations were approved by the Chinese PLA General Hospital Research Ethics Committee with written informed consent from all subjects. Human GMSCs were isolated as described in previously published protocols by Xu et al. (2014) and Moshaverinia et al. (2014). Human gingival tissues were obtained from four young adult donors (18–25 years old) who had no history of periodontal disease prior to surgery. The gingival tissues were immersed in Dulbecco's Modified Eagle Medium/Nutrient Mixture F-12 (DMEM/F12) (Gibco) containing 400 μg/ml streptomycin and 400 U/ml penicillin. The tissues were then incubated in a medium containing 2 mg/ml dispase (Gibco) at 4°C overnight after several washes with phosphate-buffered saline (PBS). Afterwards, the epithelial layer was separated from the connective tissue, and the connective tissue was minced into small fragments (1–3 mm^3^) and digested in collagenase IV (Sigma) solution at 37°C for 1 h. The retrieved cell suspension was then placed into a 10-cm cell culture dish (Corning) containing DMEM/F12 supplemented with 10% fetal bovine serum (FBS; HyClone), 2 mM L-glutamine (Gibco), 100 μg/ml streptomycin and 100 U/ml penicillin at 37°C in 5% CO_2_. Subsequently, the medium was refreshed every 3 days. After reaching 80–85% confluence, cells were detached with 0.25% trypsin-EDTA (Gibco) and counted. Cells at passages 3–8 were used in the present study.

### Surface antigen analysis and *in Vitro* multipotent differentiation of GMSCs

#### Flow cytometric analysis

Surface antigens of GMSCs were analyzed by flow cytometry. Briefly, 2 × 10^5^ cells were harvested by treatment with 0.25% trypsin-EDTA and washed twice with PBS; the cells were then incubated with a specific monoclonal antibody conjugated to either fluorescein isothiocyanate (FITC), phycoerythrin (PE), or allophycocyanin (APC) in 200 μL PBS for 30 min in the dark at 4°C. The cell surface antigens were then analyzed by flow cytometry (BD FACSCalibur, BD Biosciences). Antibodies against CD44, CD45, CD31, CD34, CD73, CD90, CD105, and CD29 (BD Biosciences) were used.

#### Osteogenic differentiation

The GMSCs were seeded in 6-well plates (1 × 10^5^ cells/well, Corning) and incubated with 2 ml of growth medium (DMEM/F12). After GMSCs reached 60% confluency, the cell medium was replaced with osteogenic induction medium: high glucose-DMEM (Gibco) containing 10% FBS, 0.1 μM dexamethasone (Sigma), 10 mM β-glycerol phosphate (Sigma), and 50 μg/ml L-ascorbic acid (Sigma). The medium was refreshed every 3 days. Two weeks later, the cells were fixed and assayed by alkaline phosphate (ALP) staining kit (Sigma).

#### Adipogenic differentiation

As described above, the GMSCs were cultured in adipogenic differentiation medium: high glucose-DMEM containing 1 μM dexamethasone, 0.5 mM 1-methyl-3-isobutylxanthine (Sigma), 100 μM indomethacin (Sigma) and 10 μM insulin (Sigma). After 2 weeks, adipogenesis was assessed by Oil Red O staining (Sigma).

#### Chondrogenic differentiation

Briefly, 1 × 10^6^ GMSCs were suspended in a 15-ml centrifuge tube at 800 g for 5 min, and the cell pellets were then cultured in chondrogenic induction medium (Cyagen Biosciences) for 3 weeks. The chondrogenic induction medium was also refreshed every 3 days. The cell pellets were then fixed, cut into 4-μm sections and stained with Alcian blue (Yang et al., 2013).

### Isolation and identification of exosomes

Exosomes were isolated and purified from the supernatant of GMSCs using the qEV size exclusion column (Izon Science) (Lobb et al., 2015; Vogel et al., 2016). Briefly, after GMSCs reached 80–85% confluency, the culture medium was replaced with fresh DMEM/F12 supplemented with 10% exosome-free FBS, and the cells were cultured for another 48 h. The supernatants were collected and centrifuged to remove death cells and cell debris and were then passed through a 0.22-μm filter. The clarified supernatant was then concentrated with 30 kDa molecular weight cut off (MWCO) hollow fiber membrane (Millipore) at 5,000 g, 4°C for 30 min. A volume of 0.5 ml of clarified supernatant was flowed through the qEV column and was eluted with PBS. The fractions from the supernatant were collected and concentrated again using the MWCO hollow fiber membrane. Final exosomes were stored at −80°C.

The protein content of the exosomes was determined using the Pierce BCA Protein Assay Kit (Thermo Fisher Scientific). The size distribution of the particles was analyzed with Tunable Resistive Pulse Sensing (TRPS, qNano, Izon Science). The morphology of the exosomes was analyzed by transmission electron microscopy (TEM, HITACHI, H-7650). Ten minutes after 10 μl of the exosomes were pipetted onto a grid coated with formvar and carbon at room temperature, excess fluid was removed, and the sample was then negatively stained with 3% phosphotungstic acid (pH 6.8) for 5 min. Finally, the samples were analyzed by TEM. The exosomal characteristic markers, CD9 and CD81 were analyzed by Western blot analysis.

### Preparation and characterization of chitosan/silk hydrogel sponge

#### Preparation of hydrogel sponge

One gram of chitosan (degree of deacetylation ≥5%, Qingdao Haihui, China) was dissolved in 50 ml 1% acetic acid solution (v/v) to prepare 2% chitosan solution. Next, 10 ml of 2% of chitosan solution was added to 10 ml of 4% silk fibroin solution and mechanically stirred at 1,500 rpm for 30 min. The hydrogel was then prepared using the freeze-drying method: the chitosan-silk-fibroin emulsion was poured into a container (basal area: 100 × 150 mm) and was incubated at −20°C for 12 h, −70°C for 6 h, and then lyophilized in a vacuum freeze dryer (UNICRYO, Germany) for 48 h to get the chitosan/silk hydrogel sponge.

#### Characterization of hydrogel sponge

The microstructure of the hydrogel sponge was evaluated by scanning electron microscopy (SEM, HITACHI, S4800). The hydrogel was immersed in distilled water, PBS, simulated body fluid (SBF) and FBS for 24 h at room temperature until swelling equilibrium state. The degree of swelling was calculated as follows:

$$\begin{array}{l} \text{Degree of swelling }=\;\left(\frac{m2-m1}{m1}\right)\times 100\% \end{array}$$

The *m*_*1*_ and *m*_*2*_ are the weights of the dry and wet hydrogel, respectively. The moisture retention capacity was evaluated by a previously described method. Briefly, after swelling equilibrium of the hydrogel, the wet hydrogel was placed in a glass dryer at room temperature, and the changes in the swelling ratio were determined every 2 h. The functional groups of the hydrogel were detected by Fourier transform infrared (FTIR) spectroscopy (Nicolet 6700).

The exosomes (50 μg) were resuspended in 50 μl PBS and loaded to a 1 × 1 cm hydrogel sponge. The presence of the exosomes on the hydrogel particles were then detected by SEM and laser scanning confocal microscopy (LSCM). The SEM images were taken according to the methods mentioned above. To detect the exosomes under the LSCM, the exosomes were labeled with the DiO cell membrane green fluorescent probe (Beyotime, China). A hydrogel with 50 μl PBS was used as a control.

### Diabetes-induced rats and wound closure assay

Twenty-four male SD rats (280–320 g) were purchased from SPF (Beijing) Biotechnology Co., Ltd. The animals were maintained at room temperature (25°C) in a 12- h light/dark cycle with free access to water and chow in the SPF environment. All the animal protocols in this study were conducted with the approval of Animal Study Committee of Chinese PLA General Hospital.

To induce diabetes, after 1 week of dietary accommodations, the rats were fed a high sucrose and high fat diet for 10 weeks and then intraperitoneally injected with STZ (35 mg/kg, Sigma) at 10 and 11 weeks. The levels of fasting blood glucose and body weight were monitored every week. Diabetes was regarded as successfully induced in rats with fasted blood glucose levels of >11 mmol/L that persisted for more than 4 weeks (Wang et al., 2017b).

Before the surgery, the rats were anesthetized by intraperitoneal injection of sodium pentobarbital (45 mg/kg). After shaving the dorsal hairs and disinfecting the skin, a 10-mm diameter full-thickness wound was created on the upper back. The rats were then randomly divided into three groups of 8 animals. Control group: the wound was covered by gauze (13 × 13 mm) containing 100 μl PBS; Hydrogel group: the wound was covered by a hydrogel (13 × 13 mm) containing 100 μl PBS; Hydrogel-Exosomes group: the wound was covered by a hydrogel after loading 100 μl PBS containing 150 μg exosomes. Afterward, a Vaseline gauze was covered the gauze (control group) or the hydrogel sponge (hydrogel and hydrogel-exosomes group). At last, restranining bandage (Urgostrapping, URGO) were used to fixed the wound and dressings. The wound dressings in each group were changed every 3 days according the above methods. At 1 week and 2 weeks post-surgery, 4 rats in each group were sacrificed for further analysis. All the wounds were photographed and the areas were measured using Image-Pro Plus 6.0 software, and the wound closure rates were then calculated: wound closure rate = (A_0_ − A_t_)/A_0_. A_0_ is the initial wound area, and A_t_ is the wound area at 1 week or 2 weeks post-surgery.

### Histology analysis

Four wound areas, including the surrounding healthy skin in each group, were excised and studied by histopathological analysis at 1 week and 2 weeks after surgery. The excised skin was fixed in 4% paraformaldehyde, gradually dehydrated, embedded in paraffin and sliced into 4-μm-thick sections. The sections were stained with hematoxylin and eosin (H&E) staining and the lengths of the neo-epithelium were calculated according to previously described methods (Zhang et al., 2015c, 2017). Masson's trichrome staining was used to determine the content and maturity of collagen in the wound beds. The fraction of collagen was calculated by detecting the blue area under the 400× magnification fields of each group (four files randomly in each group) using Image-Pro Plus 6.0 software.

### Immunohistochemical analysis

Angiogenesis in each group was determined by staining of CD34 in the wound bed. The sections were treated by antigen retrieval and then incubated with primary antibody (rabbit anti rat, Abcam) at 4°C overnight. Subsequently, Antibody binding of tissue sections were visualized by incubating with DAB substrate. The CD34 staining for blood vessels was observed under 400× magnification. For each slide, the microvessel density was calculated as the number of CD34-positive microvessels in four random fields (400×) using Image-Pro Plus 6.0 software.

### Immunofluorescence analysis

The neuronal ingrowth at 2 weeks post-surgery was evaluated by immunofluorescent staining of 200 kDa neurofilament heavy chain (NEFH, rabbit anti-rat, Abcam) in the wound bed. Briefly, the tissue sections were blocked in 1% BSA for 30 min at room temperature, incubated with primary antibody overnight at 4°C and then incubated with secondary Alexa Fluor® 647 antibody (goat anti-rabbit, Abcam) for 1 h at room temperature. In addition, 2-(4-Amidinophenyl)-6-indolecarbamidine dihydrochloride (DAPI; Beyotime) was used to stain the cell nuclei. The area of red fluorescence in different groups was determined by counting four random fields per section using Image-Pro Plus 6.0 software.

### Statistical analysis

All the data were expressed as the mean ± standard deviation (SD). One-way analysis of variance (ANOVA) and Student-Newman-Keuls (SNK) *post-hoc* tests were applied to compare the differences between groups using SPSS 19.0 software. *P* < 0.05 were considered statistically significant.

## Results

### Characterization of GMSCs and *in Vitro* multipotent differentiation

**Figure 2:**
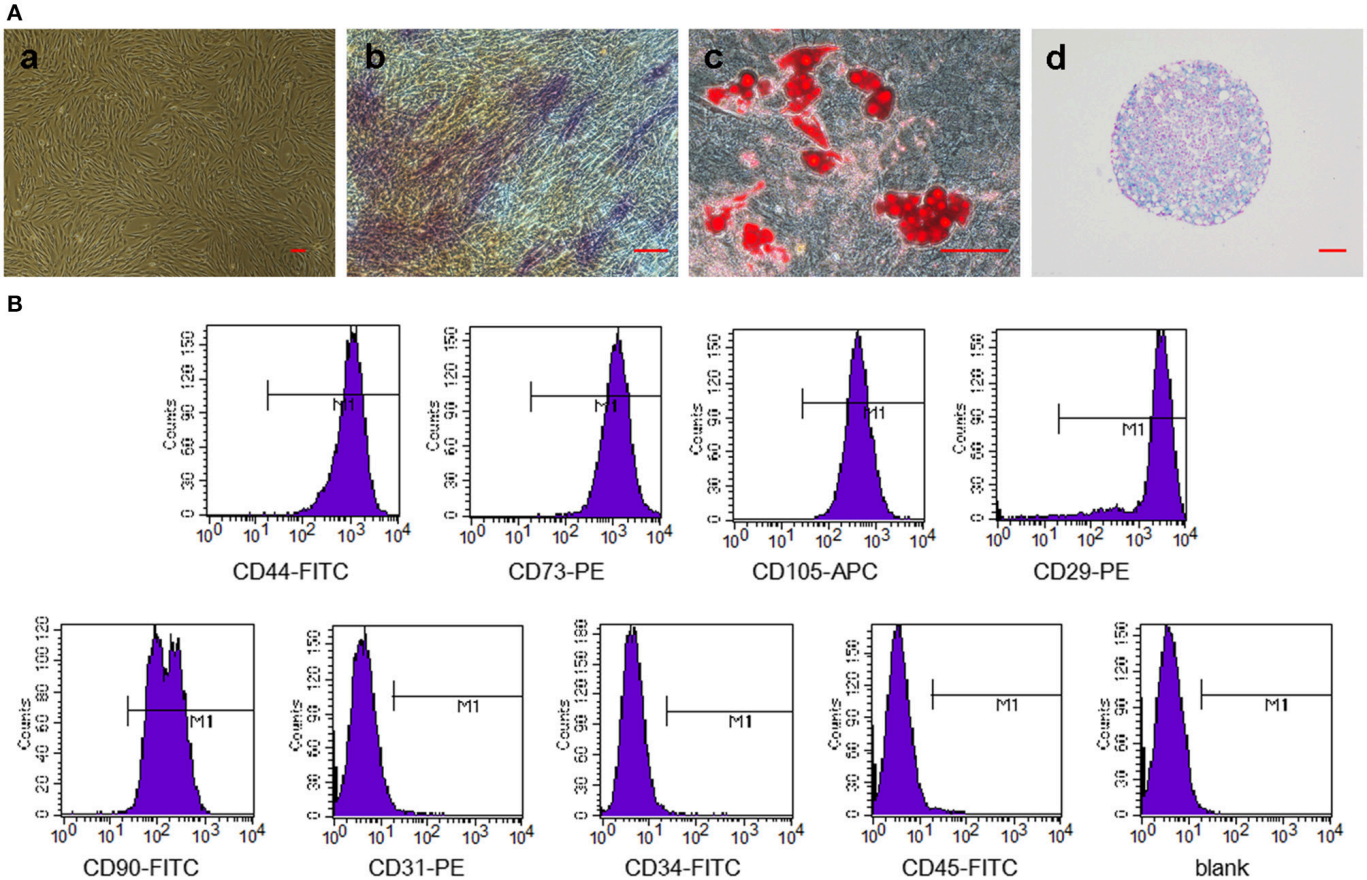
Characterization of GMSCs and *in vitro* multipotent differentiation. **(A)** Representative images of GMSCs at passage 3 **(a)**. Representative images of osteogenesis **(b)**, adipogenesis **(c)**, and chondrogenesis **(d)** of GMSCs stained with alkaline phosphatase, Oil Red O and Alcian blue, respectively. Scale bar: 100 μm. **(B)** Flow cytometric analysis of surface markers in GMSCs.

We have successfully derived GMSCs as shown in Figure 2Aa, and the cells at passage 3 exhibited a spindle-like morphology *in vitro*. The results of tri-lineage differentiation experiments demonstrated the multi-potency of the GMSCs (Figures 2Ab,c,d). Flow cytometry analysis revealed that GMSCs positively expressed MSC markers (Figure 2B), CD44 (99.99%), CD73 (100%), CD90 (99.87%), CD105 (100%), and CD29 (99.53%) and were negative for hematopoietic stem cell markers, CD31 (0.75%), CD34 (0.17%), and CD45 (0.54%). These results were consistent with previous studies.

### Characterization of GMSC-derived exosomes

**Figure 3:**
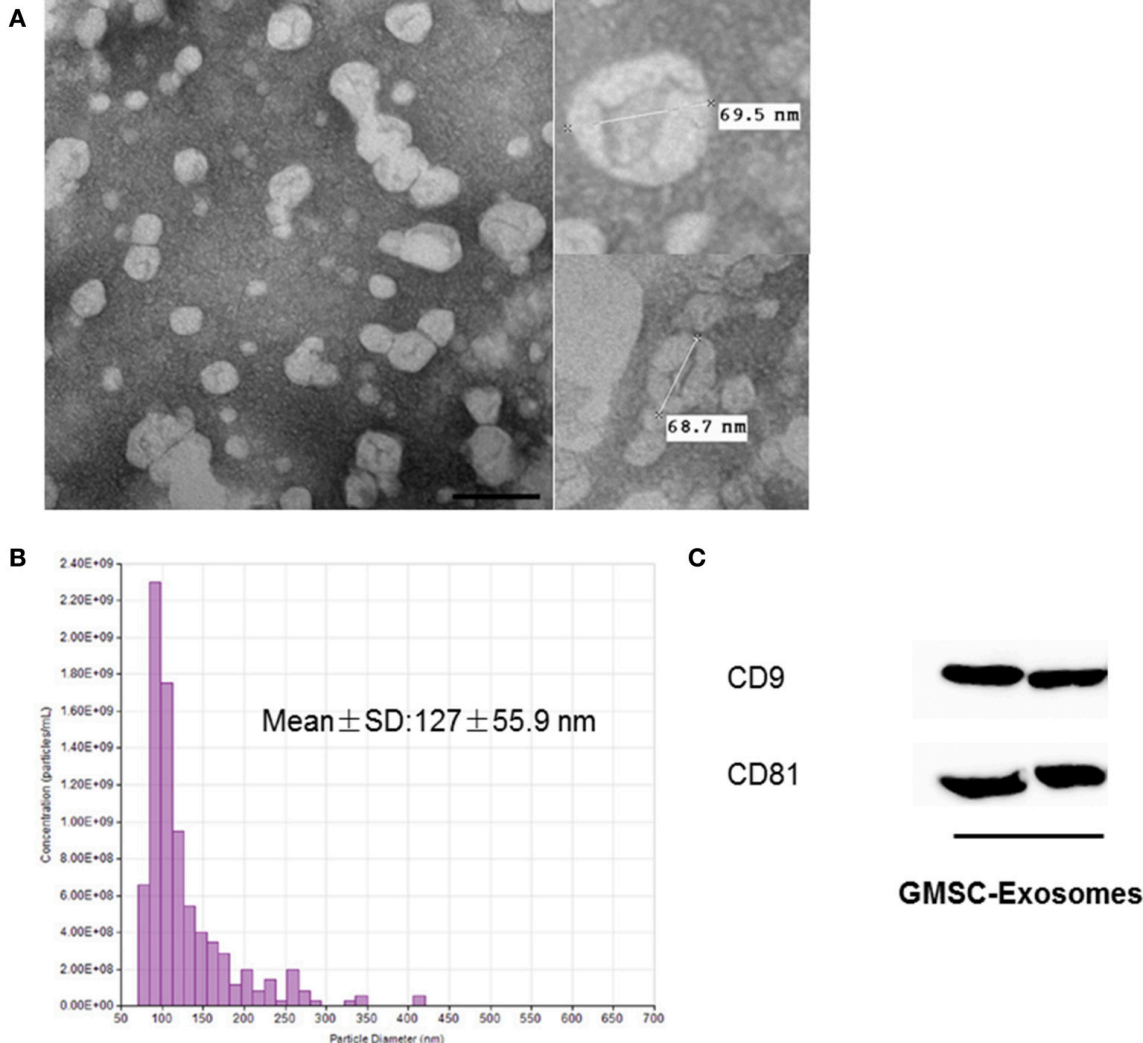
Characterization of GMSC-derived exosomes. **(A)** Representative images of the morphology of GMSC-derived exosomes by TEM (scale bar: 100 nm). **(B)** TRPS analysis demonstrates exosomes have a single peak (~80 nm) diameter, and the mean diameter is 127 nm. **(C)** Detection of exosomal marker (CD9 and CD81) expression in exosomes by Western blot analysis.

GMSC-derived exosomes were successfully isolated using the method we previously described. As shown in Figure 3A, the exosomes were observed as spherical structures by TEM. Figure 3B shows the histogram of the TRPS analysis, which revealed that exosomes have a single peak (~80 nm) diameter and the mean diameter of our isolated exosomes is 127 ± 55.9 nm. The results of Western blot analysis showed that GMSC-derived exosomes expressed the exosomal markers CD9 and CD81 (Figure 3C).

### Characterization of the chitosan/silk hydrogel sponge

**Figure 4:**
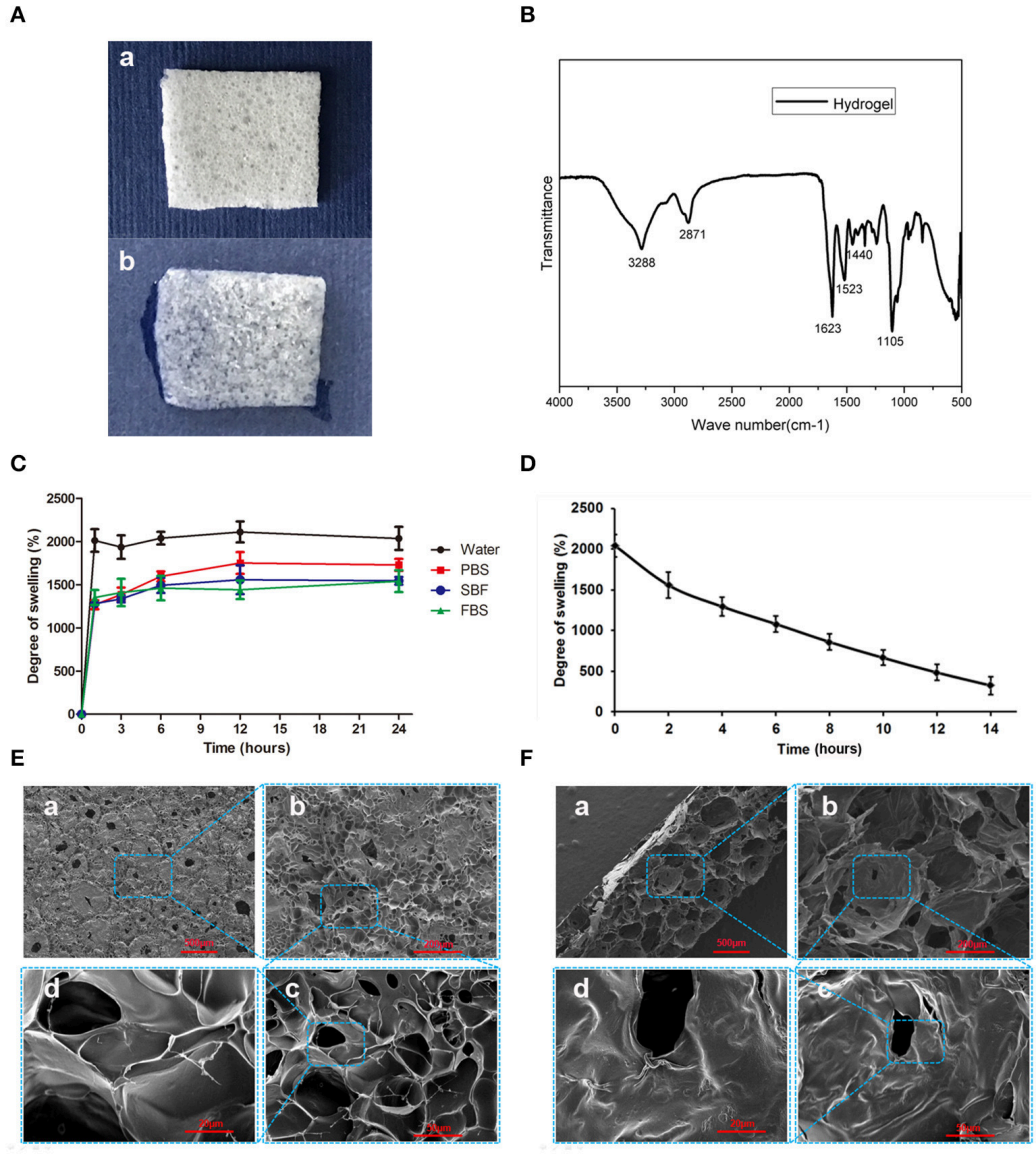
Characterization of the chitosan/silk hydrogel sponge. **(A)** Morphology of dry hydrogel sponge **(a)** and wet hydrogel sponge **(b)**. **(B)** FTIR spectra of the chitosan/silk hydrogel sponge. **(C)** Swelling degree of the hydrogel in different medium at different time. **(D)** Moisture retention capacity of the hydrogel sponge. **(E)** SEM images of hydrogel surface (**a**: 30×; **b**: 100×; **c**: 400×; **d**: 1,000×) **(F)** SEM images of cross-sections of hydrogel (**a**: 30×; **b**: 100×; **c**: 400×; **d**: 1,000×).

Figure 4A demonstrates the morphology of the hydrogel is a thin porous sponge. The FTIR spectrum of the chitosan/silk hydrogel (Figure 4B) exhibited several characteristic bands: the vibration of N-H and O-H groups is located ~3,288 cm^−1^; the bands at 2871 cm^−1^ correspond to the C-H stretching vibration in the –CH and –CH_2_ groups; the peak at 1623 cm^−1^ corresponds to the C = O stretching; N-H bending vibration overlapping amide II vibration is at 1523 cm^−1^; the C-H deformation vibration is located at 1,440 cm^−1^; and the peak at 1105 cm^−1^ has been assigned to the C-O in stretching. Figure 4C shows the swelling properties of the chitosan/silk hydrogel in different medium at different time points. After 12 h, the swelling equilibrium was reached in each solution, and the water absorbing capacity of the hydrogel was found to be almost 20 times of its weight. In addition, in PBS, SBF and FBS, the swelling ratio was 17, 15, and 14, respectively. In addition, the hydrogel also exhibited good moisture retention capacity (Figure 4D). The water retention time was more than 12 h and there are 484% of their-own-weight water residues in the hydrogel 12 after hours.

The SEM images in Figure 4E show that the surface of the hydrogel sponge has many pores ranging in size from 50 to 150 μm. In addition, the pore wall surface appears smooth and thick. Figure 4F shows the cross-section micrograph of the hydrogel sponge. Larger pores are observed as ranging in size from 200 to 500 μm.

**Figure 5:**
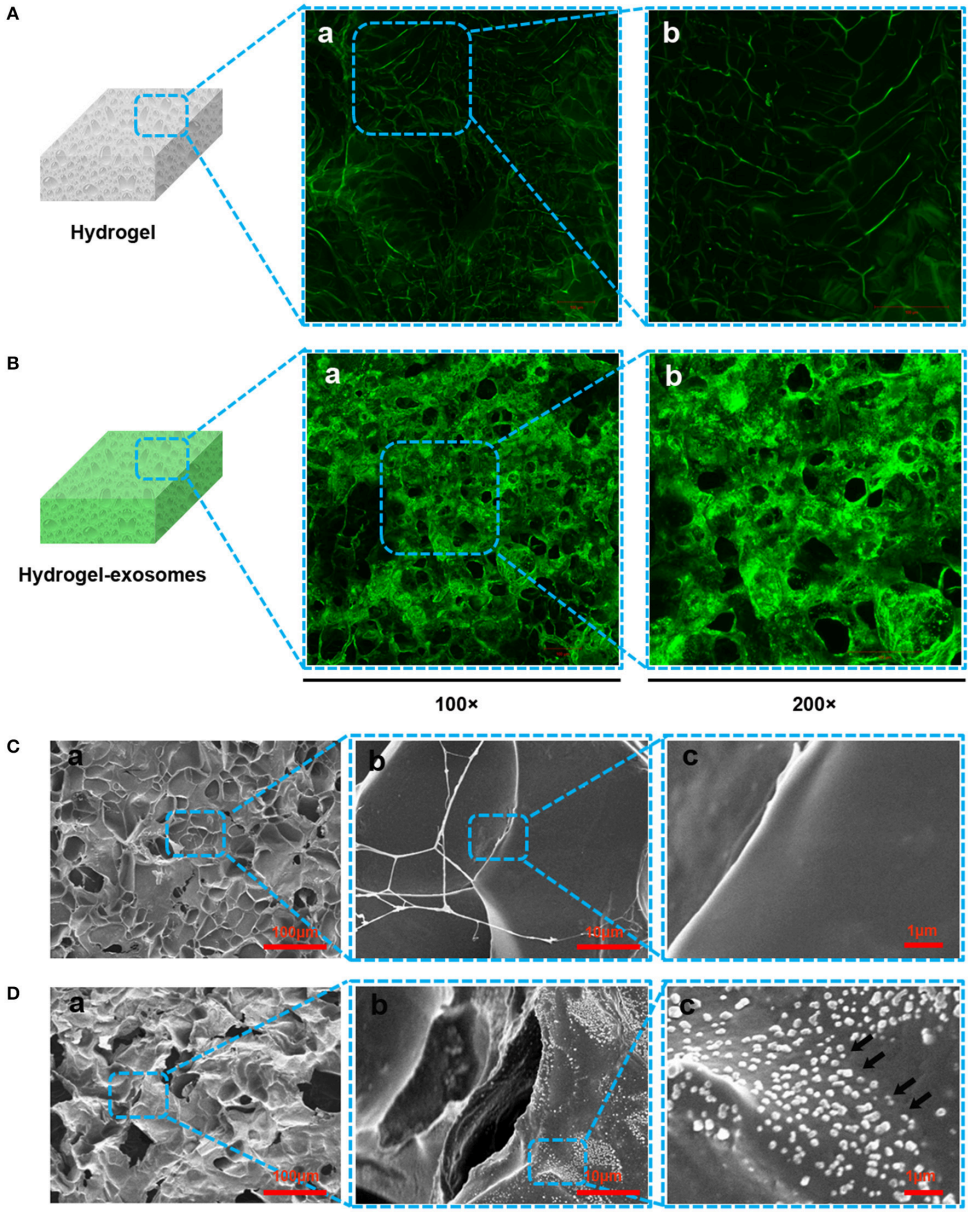
Detection of exosomes on the hydrogel sponge. **(A)** LSCM images of the hydrogel sponge. (**a**: 100×; **b**: 200×). **(B)** LSCM images of the hydrogel sponge with DiO-labeled exosomes. (**a**: 100×; **b**: 200×). Scale bar: 100 μm. **(C)** SEM images of hydrogel surface (**a**: 100×; **b**: 1,000×; **c**: 6,000×). **(D)** SEM images of hydrogel surface after adding the exosomes (**a**: 100×; **b**: 1000×; **c**: 6000×). The black arrows show the exosomes.

According to the LSCM images, compared to the control chitosan/silk hydrogel (Figure 5A), we detected more green fluorescence in the hydrogel after loading the DiO-labeled exosomes (Figure 5B), which confirmed the presence of the exosome particles. In the SEM images, the surface morphology and structure of the hydrogel sponge after adding the exosomes was not noticeably different. In the low-resolution images, compared with the control hydrogel (without exosomes, Figure 5Ca), the hydrogel adding exosomes give the impression of coarseness and grittiness (Figure 5Da). In the high-resolution images (Figures 5Db,c), exosome particles can be observed on the hydrogel sponge (black arrows in Figure 5Dc).

### Evaluation of *in Vivo* wound closure using chitosan/silk hydrogel and hydrogel-exosomes in diabetic rats

After the injection of STZ, the fasting blood glucose tests revealed that the blood glucose levels of all the rats were higher than the 11 mmol/L (19.65 ± 4.15) observed at 12 weeks, and this condition was stable for the following 4 weeks. In addition, the typical symptoms of body weight-loss, polyphagia, polydipsia, and polyuria after the injection of STZ were observed in the rats. Compared with normal rats, the H&E staining of the pancreatic tissue in the diabetic rats confirmed the pathological changes in STZ-treated rats. The acinar cells were swollen and the islet β-cells were damaged (Figure S1). All these results indicated that we have successfully induced a stable diabetes model.

**Figure 6:**
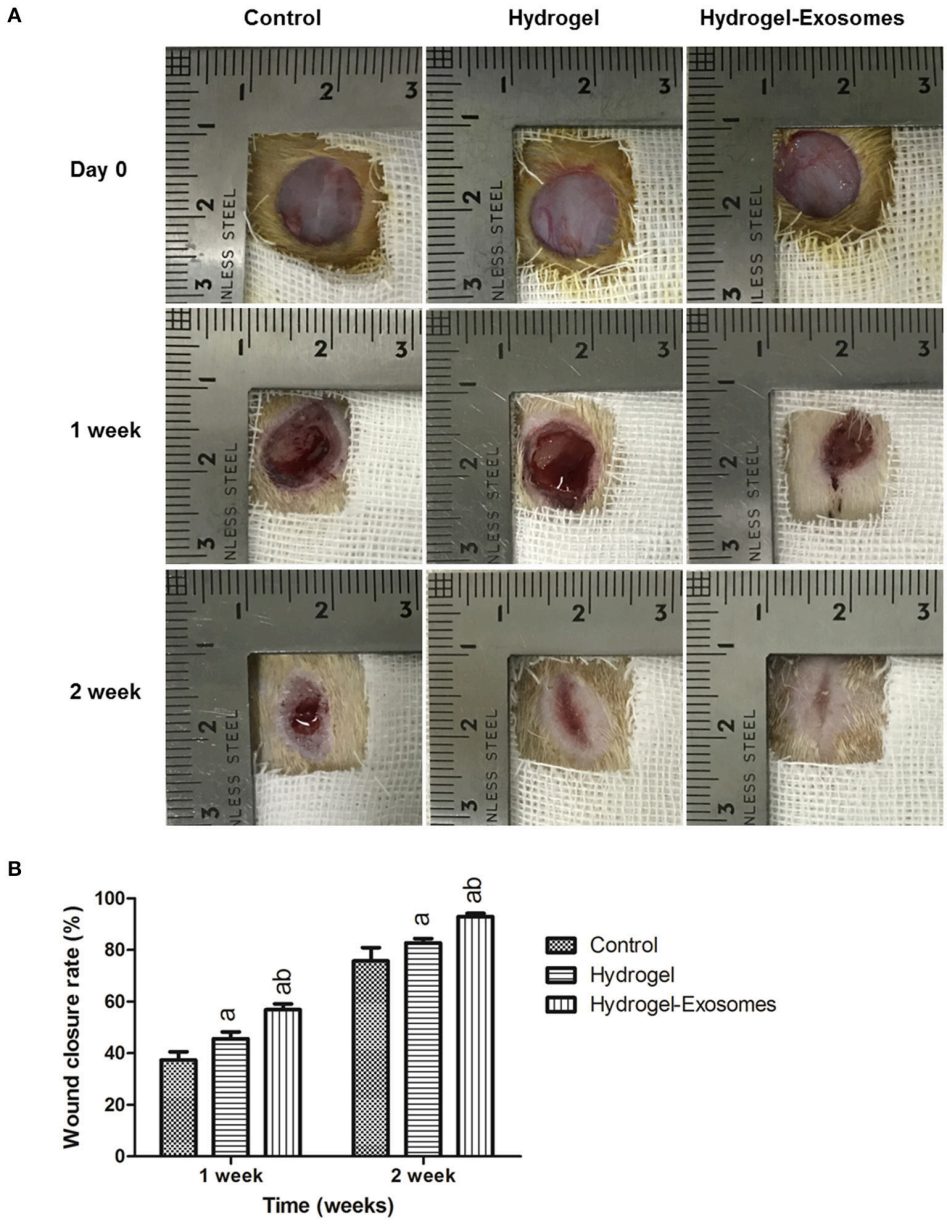
Macroscopic appearances and quantitative analysis of cutaneous wounds in the different groups. **(A)** Representative images of full-thickness skin defects in diabetic rats at 0, 1 week, and 2 week post-surgery of the control group, hydrogel group and hydrogel-exosomes group. **(B)** Quantitative analysis of the wound closure rates in each group at 1 week and 2 weeks post-surgery (*n* = 4 in each group). a, *P* < 0.05 compared to the control group; b, *P* < 0.05 compared to the hydrogel group.

Figure 6A shows optical images of the control group, hydrogel group and the hydrogel-exosomes group at 0, 1 week, and 2 weeks post-surgery. While the wound size in all the groups decreased with time, the wound size of the hydrogel-exosomes group was smaller than the other two groups, and the wounds had almost closed by 2 weeks. In addition, the hydrogel groups had a better repair effect than the control group. Quantitation of the wound size confirmed the above results (Figure 6B). At 1 and 2 weeks post-surgery, the hydrogel and hydrogel-exosome group showed significantly better healing effects compared to the control group. While the hydrogel-exosomes group showed significantly better healing effects than the hydrogel group.

### Histologic analysis cutaneous wounds healing in the different groups

**Figure 7:**
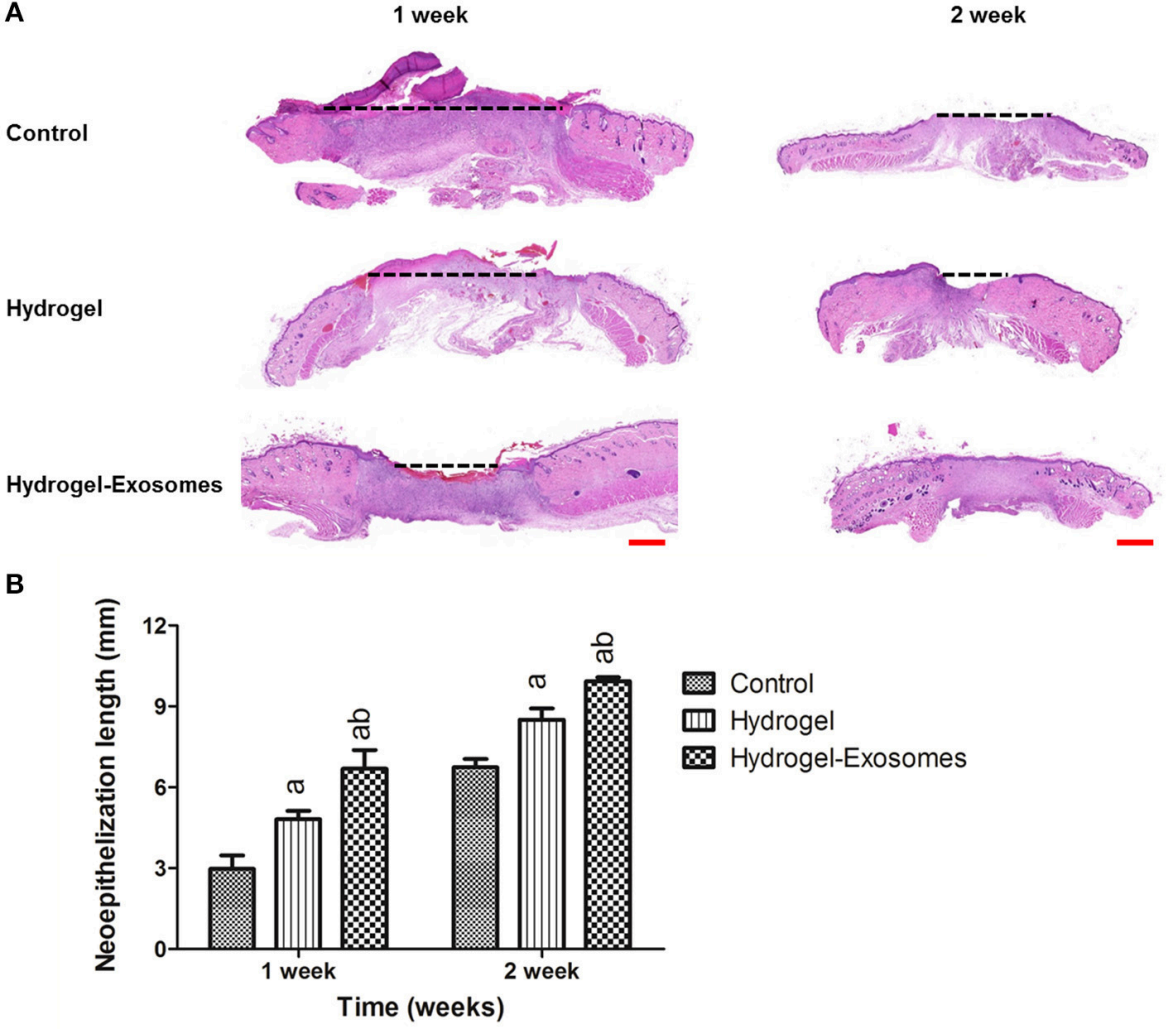
H&E staining of the wound sections and quantitative analysis of the neo-epithelium. **(A)** Representative images of H&E staining of the wound section in each group at 1 week and 2 weeks post-surgery. The black dotted line indicates the length without re-epithelialization in the wound. Scale bar: 1 mm. **(B)** Quantitative analysis of the total length in the three groups at each time point. a, *P* < 0.05 compared to the control group; b, *P* < 0.05 compared to the hydrogel group.

The H&E stained sections showed the neo-epithelium in the wound defects in the three groups (Figure 7A). The black dotted line indicated the length without re-epithelialization in the wound. The total neo-epithelium in the hydrogel-exosomes group was significantly longer than that in the hydrogel group or the control group at 1 week and 2 weeks post-surgery. While the neo-epithelium length of the wound defects in the hydrogel group was significantly longer than the control group at 1 week and 2 weeks (Figure 7B).

**Figure 8:**
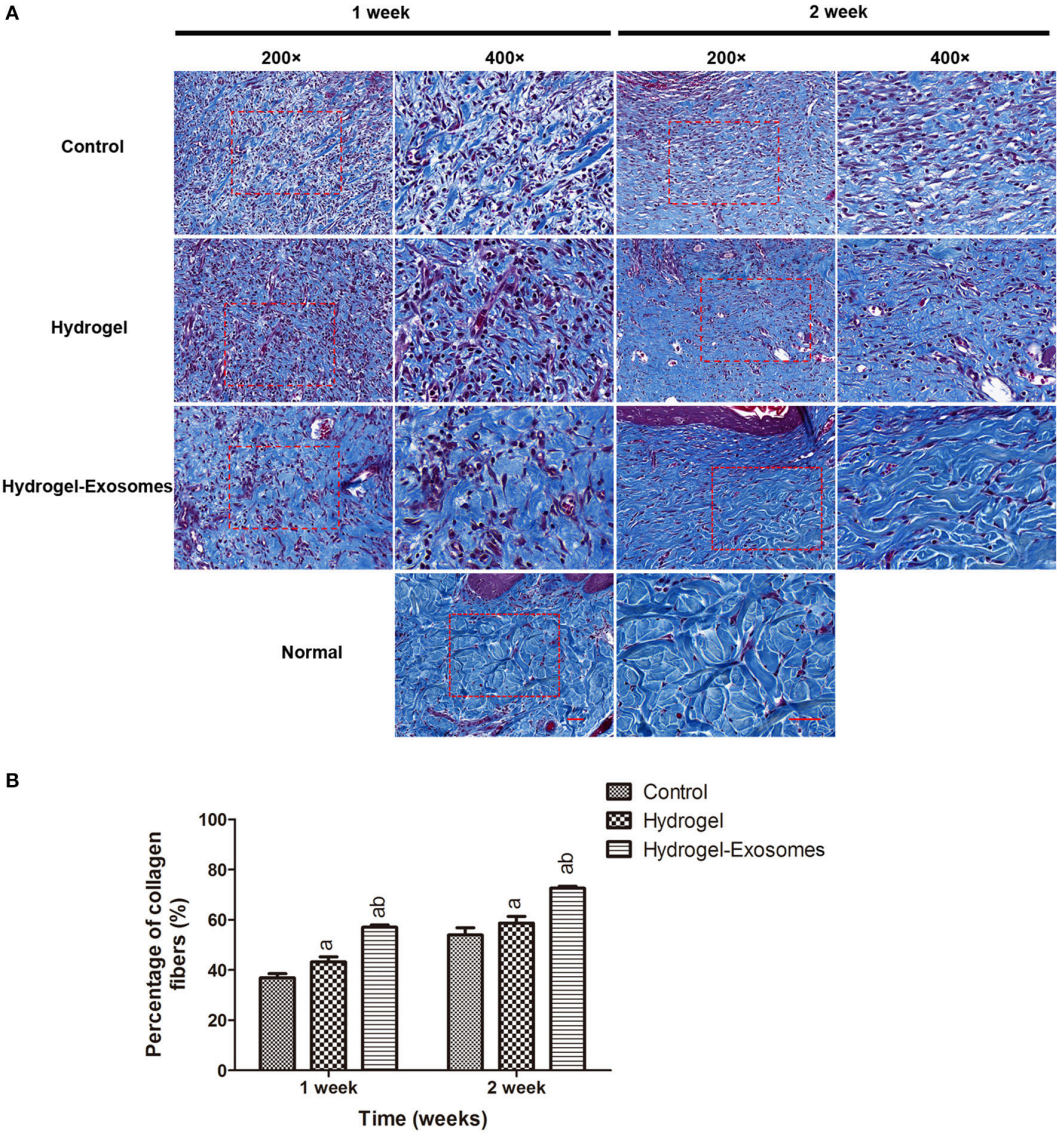
Masson's trichrome staining of wound sections and quantitative analysis of collagen deposition. **(A)** Representative images of Masson's trichrome staining of the wound section in each group at 1 week and 2 weeks post-surgery. As a control, the normal skin is also shown. The 400× image is the magnified view of the area denoted by the dashed boxes in the 200× image. Scale bar: 50 μm. **(B)** Quantitative analysis of the percentage of collagen in each group at each time points. a, *P* < 0.05 compared to the control group; b, *P* < 0.05 compared to the hydrogel group.

Masson's trichrome staining and quantitative analysis were applied to evaluate the ECM deposition and maturation. As shown in Figure 8A, at 1 week post-surgery, immature collagen could be seen surrounding the skin fibroblasts in the hydrogel and control groups. Extensive deposition of collagen fibers was observed in the wound bed of the hydrogel-exosome group compared with the other two groups at 1 week and 2 weeks post-surgery, and the difference was statistically significant. Moreover, at 2 weeks post-surgery, there was more collagen deposition and thick wavy collagen fibers in the hydrogel-exosomes group, and the collagen fibers arranged in an orderly fashion, which is similar to that of normal skin. Quantitative analysis (Figure 8B) revealed the content of the collagen in the hydrogel-exosome group and hydrogel group was significantly higher than the control group, and the collagen content of the hydrogel-exosome group was significantly higher than the hydrogel group.

### Immunohistochemical analysis of microvessel density

**Figure 9:**
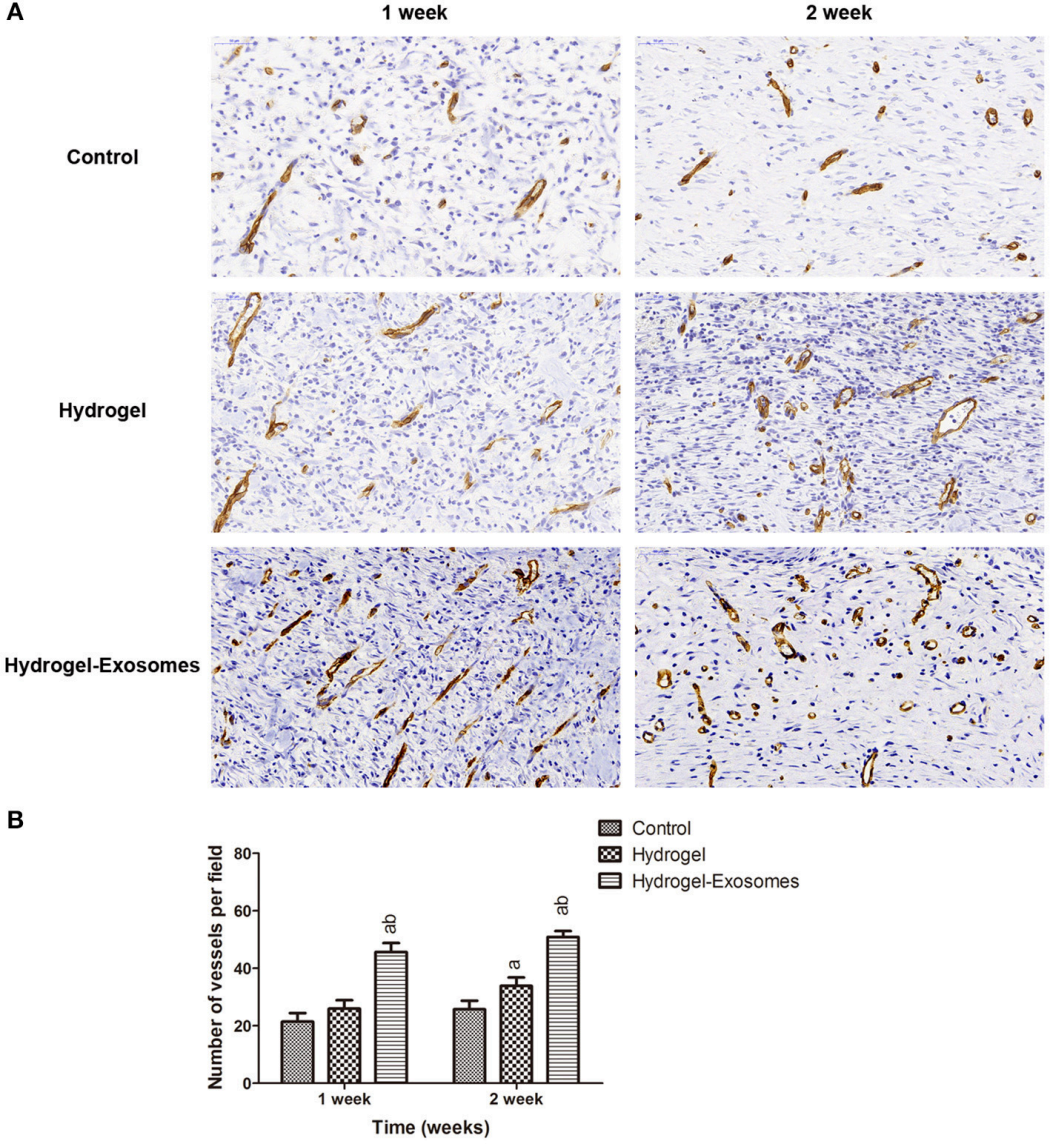
Immunohistochemical analysis of angiogenesis. **(A)** Representative images of immunohistochemical staining of CD34 in each group at 1 week and 2 weeks post-surgery (400×). Scale bar: 50 μm. **(B)** Quantitative analysis of the number of microvessels per field in each group at 1 week and 2 weeks post-surgery. a, *P* < 0.05 compared to the control group; b, *P* < 0.05 compared to the hydrogel group.

Immunohistochemical staining of CD34 was performed to detect angiogenesis in the wound bed at 1 week and 2 weeks post-surgery. As shown in Figure 9A, more neogenetic microvessels were observed in the hydrogel-exosomes group at 1 week post-surgery and were arranged in parallel. While at 2 weeks, more typical round- or oval-shaped microvessels were observed in this group. Quantitative analysis of the microvessels showed an increase in the number of microvessels from 1 week to 2 weeks post-surgery for all three treatment groups (Figure 9B). While in the hydrogel-exosome group, the number of the microvessels was significantly higher than the hydrogel group and control group. The number of the microvessels in the hydrogel group was higher than the control group at 2 weeks post-surgery.

### Immunofluorescent analysis of nerve fiber density at 2 weeks post-surgery

**Figure 10:**
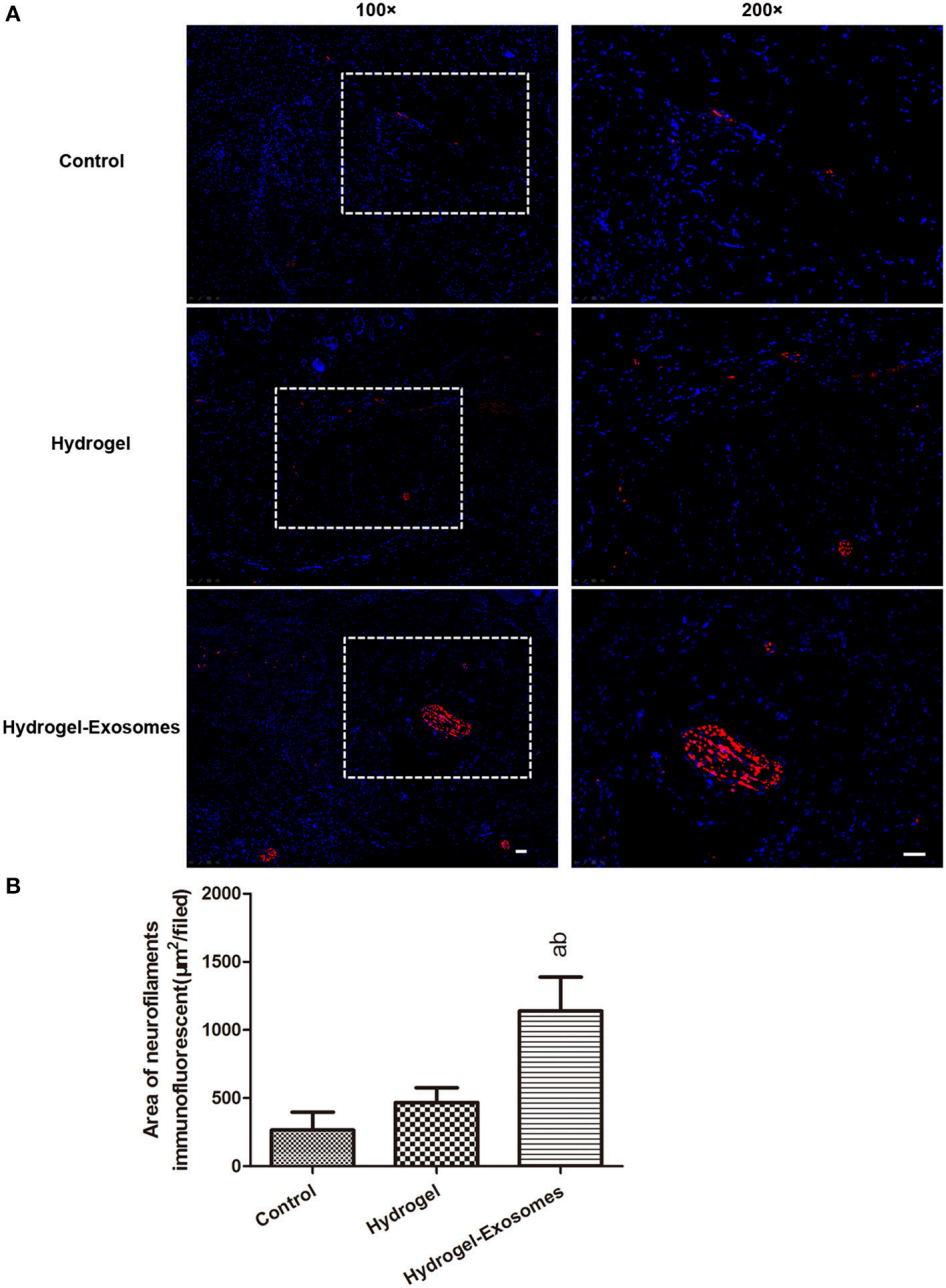
Immunofluorescent analysis of nerve fiber density at 2 weeks post-surgery. **(A)** Representative immunofluorescent images of neurofilament heavy chain (red fluorescence) were detected at 2 weeks post-surgery. Nuclei were stained with DAPI (blue fluorescence). The 200× image is the magnified view of the area denoted by the dashed boxes in the 100× image. Scale bar: 50 μm. **(B)** Quantitative analysis of the nerve fiber density in each group at 2 weeks post-surgery. a, *P* < 0.05 compared to the control group; b, *P* < 0.05 compared to the hydrogel group.

To assess nerve fiber growth, neurofilaments were detected by anti-neurofilament heavy polypeptide antibody and indicated as red fluorescence at 2 weeks post-surgery (Figure 10A). The results revealed that the nerve fiber density was significantly increased in the hydrogel-exosomes group compared with the other two groups. Although the nerve density was higher in the hydrogel group than in the control group, the difference was not statistically significant (Figure 10B).

## Discussion

Because diabetic chronic wounds, such as diabetic foot ulcers (DFUs), are one of the leading factors that threaten the mental and physical health of DM patients and cause economic burden, the research and development of an ideal treatment method is both important and urgent (Sener and Albeniz, 2015; Cao and Gang, 2017). In the present study, our combination of GMSC-derived exosomes and a chitosan/silk hydrogel sponge was effectively fabricated to accelerate skin defect healing in a diabetic rat model.

Although stem cell-based therapies have shown beneficial effects on wound healing and tissue regeneration, there have been reports that the predominant mechanism of stem cells in repairing tissue results from paracrine action instead of transdifferentiation (Togel et al., 2007; Zhang et al., 2015b). Increasingly more studies have proven that the paracrine functions of stem cells could be mediated by exosomes, which have significant potential as a novel alternative to whole cell-based therapy and to achieve “cell-free regenerative medicine” (Kourembanas, 2015; Rani and Ritter, 2016; Phinney and Pittenger, 2017). Stem cell-derived exosomes contain cytokines and growth factors, signaling lipids, mRNAs, and regulatory miRNAs (Yáñez-Mó et al., 2015). Moreover, the application of the exosomes may have a superior safety profile and could overcome the allogeneic and xenogeneic immunological rejection and oncogenic mutations in cell transplantation therapy (Rani et al., 2015). As such, we chose to use exosomes to repair skin wounds in the current study.

GMSCs have many advantages, such as GMSCs are homogeneous, non-tumorigenic, easily isolated, and phenotypically stable (Cao and Gang, 2017). In the present study, we successfully isolated GMSCs from human gingival tissue, which showed osteogenic, adipogenic and chondrogenic differentiation capabilities. Moreover, the isolated GMSCs expressed specific surface antigens: CD44, CD73, CD90, CD105, and CD29, and did not express hematopoietic antigens: CD31, CD34, and CD45. These results were consistent with previous reports. (Moshaverinia et al., 2014; Xu et al., 2014) Zhang et al. and Jiang et al. have proven that the GMSCs could accelerate skin wound healing by enhancing re-epithelization, collagen deposition, and angiogenesis in the wound bed and by inhibiting the production of pro-inflammatory cytokines and increasing anti-inflammatory cytokines (Zhang et al., 2010; Jiang et al., 2015). These results indicated the GMSCs may play a critical role in skin wound healing; however, to our knowledge, there is no study showing the isolation and characterization of GMSC-derived exosomes in this field. In our study, using a qEV isolation kit, we have successfully isolated and characterized exosomes from GMSCs.

Accumulating evidence indicates that stem cell-derived exosomes, for example, human umbilical cord MSCs (hUCMSCs) (Zhang et al., 2015b), induced pluripotent stem cells (iPSCs) (Zhang et al., 2015c), and adipose mesenchymal stem cells (ASCs) (Hu et al., 2016), could effectively promote wound healing. In most of these studies, the exosomes were isolated and applied through subcutaneous injection to several sites around the wound. By this method, the exosomes did not have direct contact with the injury site. Because transwell assays in many studies revealed that exosomes can attract fibroblasts and endothelial cells, both of which play a critical role in wound healing (Zhang et al., 2015a,b), direct contact with the wound may have greater beneficial effects. Because of the neuropathy and peripheral vascular disease in DM patients, even a small wound is likely to lead to non-healing ulcers and increase the risk of bacterial infections, which may put patients at risk of additional severe complications, especially in the extremities (hands and feet) (Greenhalgh, 2003; Brem et al., 2004). Therefore, in the current study, we designed a porous hydrogel sponge that we hope not only serves as a wound dressing but also serves as a non-invasive method to deliver exosomes directly to the wound. The chitosan/silk hydrogel sponge showed good swelling behavior, as chitosan is a hydrophilic polymer and swelling equilibrium could be achieved at 12 h. It is widely accepted that a moist environment could promote wound healing by accelerating re-epithelialization (Korting et al., 2011). In this study, the hydrogel has good moisture retention capacity. The SEM images showed that the hydrogel maintained an interconnected microporous structure. In this study, all these characteristics suggest the hydrogel/silk hydrogel sponge is a very suitable option for wound dressing and can serve as a suitable scaffold for exosomes.

After the loading of the exosomes to the hydrogel, we can see many spherical exosome particles on the surface of the hydrogel without affecting the morphology of the hydrogel. After application of the hydrogel-exosomes to the wound of diabetic rats, our results demonstrated that this method greatly promoted the skin wound healing, and at 2 weeks post-surgery, the wounds were almost healed. Because the healing effect in the hydrogel-exosomes group is faster than the hydrogel group, we speculate these results have two implications: first, the application of GMSC-derived exosomes can promote the healing of the diabetic skin wound; second, this non-invasive method of exosomes delivery to the wound could also achieve the biological effects of the exosomes. Moreover, compared with the control group, the wound size was smaller in the hydrogel group, which confirmed that the hydrogel alone also have beneficial effects on wound healing. The healing effects may be contributed to many factors, i.e., the structure and physical properties of the hydrogel, and the beneficial effects of the chitosan and silk (Dai et al., 2011; Kapoor and Kundu, 2016).

As the largest organ in the human body, the skin is composed of two primary layers, the epidermis and dermis. The epidermis provides a barrier in preventing pathogens from entering and retaining water in the body, while the dermis is essential for achieving functional and aesthetic outcomes. Previous studies have found that MSC-derived exosomes could promote the re-epithelialization of skin wounds by inducing epithelial cell proliferation and activating collagen secretion by fibroblasts (Zhang et al., 2015a,c). In our study, the hydrogel-exosome group exhibited longer neo-epithelium in the H&E staining and a large improvement in the collagen deposition and mature in the Masson's staining. At 2 weeks post-surgery, the defect sites in the hydrogel-exosomes group were almost covered by neo-epithelium with more arranged, orderly, wavy collagen fibers, which is similar to the normal skin. These results indicated the positive influence of the GMSC-derived exosomes on the re-epithelialization and the deposition and remodeling of the ECM.

For the diabetic patients, microvascular disease is a main factor that contributes to delayed or non-healing of the wound, which suggests that a decrease in the supply of nutrients and oxygen to the surrounding tissue reduces the ability to fight infection (Greenhalgh, 2003). As shown in Figure 6A, skin defects in our diabetic rat model resulted in almost no bleeding in the wound site. Therefore, one of the critical components of wound healing is the formation of the new microvascular system. Several studies have found that MSC-derived exosomes can increase angiogenesis in wound healing models and promote the function of the human umbilical vein endothelial cells (HUVECs). (Zhang et al., 2015c; Liang et al., 2016) In our studies, to determine the effects of different treatments on skin angiogenesis, immunohistochemical staining of CD34 was conducted to quantify the microvessels in the wound bed. The results confirmed the increase in microvessel number in the wound bed of the hydrogel-exosomes group. The higher microvessel density in the wound bed indicates a sufficient supply of oxygen and nutrients, and all these factors can enhance the healing of the skin defects in diabetic rats.

Diabetic neuropathy has also been reported as a contributor to the development of problem wounds in DM patients, especially in the lower extremities, which is observed in 50% of DFU patients (Greenhalgh, 2003; Pop-Busui et al., 2016). Neurofilaments are key cytoskeletal structures for neurons and axons(Zochodne et al., 2004), thus the immunofluorescent staining of NEFH were applied in our study to evaluate nerve regeneration at 2 weeks post-surgery. Compared to the hydrogel group, the addition of GMSC-derived exosomes significantly promoted the nerve density, which suggests that the GMSC-derived exosomes could facilitate neuronal ingrowth to the wound bed. Previous studies have found that MSCs could promote nerve growth, restoration of neuropathic morphology and nerve function in diabetic rats, and the main mechanism is the secretion of nerve growth factor (NGF) by the MSCs, which can in turn promote the regeneration of nerve fibers (Sun et al., 2012; Xia et al., 2015; Wu et al., 2016). However, to our knowledge, there are no studies focused on the effects of MSC-derived exosomes on neuronal growth in diabetic rat skin defect model at present, and our results provide new evidence that the use of MSC-derived exosomes may be a promising method to promote the healing of chronic wounds in DM patients.

Previously, we have successfully composite porous scaffolds with PLGA particles loading insulin to achieve bone restoration in rabbits critical size bone defect (Wang et al., 2017a). Similarly, in current study, the chitosan/silk hydrogel loading GMSC-derived exosomes revealed positive effects on the healing of skin defects in the diabetic rats. In addition, this study provided a novel concept for the application of exosomes. Since it has been proven that stem cell-derived exosomes play a critical role in tissue repair and regeneration, some researchers began to explore new methods to apply exosomes in the tissue repair field. Different from the traditional injection methods, the use of exosomes combined with biomaterials is the major focus of tissue repair research. Liu et al. prepared a photo induced imine crosslinking hydrogel glue to act as an exosome scaffold (Liu et al., 2017). The results revealed that hydrogels can retain stem cell-derived exosomes and effectively promote the repair and regeneration of articular cartilage defects. To achieve controlled release of the exosomes to the wound sites, Tao et al. added the exosome directly to the chitosan solution to prepare a hydrogel (Tao et al., 2016). This method also promoted the wound healing of a rat skin defect model. In our study, the preparation of the hydrogel and addition of exosomes are two separate processes because we wanted to avoid the acetic acid impairment of the exosomes and shorten the extracellular exposure of the exosomes. Considering the tissue repair ability of the exosomes, more application methods of exosomes will be explored and adapted in the future.

Despite our results revealed that the combination of the hydrogel and exosomes have beneficial effect on the diabetic rat skin wound repair, we think there are also some limitations about our animal model and experiments. First, the animal model has only been proved by the H&E staining of the pancreatic tissue and the blood glucose. As we all know, the diabetes chronic wound is also commonly combined with alternations in inflammatory factors, microvascular disease and neuropathy, so more experiments to verified these characteristics or a comparison with non-diabetic rats may give our results more arguments. Second, despite rats has been widely used as skin defect model for preclinical research, the inherent characteristics of wound healing in rats is different from human, therefore a pig model maybe more suitable and more reliable.

## Conclusion

In conclusion, we have successfully isolated and characterized the GMSC-derived exosomes. The chitosan/silk hydrogel sponge, prepared using the freeze-drying method, has desirable structural and physical properties that can be beneficial for wound healing and can be used as a scaffold for the exosomes. The combination of the exosomes and hydrogel could effectively promote the skin wound healing in an STZ-induced diabetic rat model by promoting the re-epithelialization, deposition and remodeling of ECM and by enhancing angiogenesis and neuronal ingrowth. These findings not only provide new information on the role of the GMSC-derived exosomes in wound healing but also provide a novel non-invasive application method of the exosomes with practical value for skin repair. Further, we will continue to explore the exact mechanism of how GMSC-derived exosomes enhance skin regeneration.

## Author contributions

Experimental work performance and manuscript drafting: QS and ZQ; Data collection and related analysis: DL and JS; Data analysis and manuscript revise: XW, XG, and JX; Study design and coordinating experiment: HL, JX, and XG. All authors read and approved the final manuscript.

### Conflict of interest statement

The authors declare that the research was conducted in the absence of any commercial or financial relationships that could be construed as a potential conflict of interest. The reviewer FR and handling Editor declared their shared affiliation.

## Supplementary material

Figure S1 H&E staining of pancreatic tissue in normal and diabetic rats. **(A,B)** Normal architecture of the pancreas in the control rat. The black arrows show the islet cells. **(C,D)** The section of the diabetic rat pancreas displayed pathological changes. The acinar cells were swollen and the islet β-cells were damaged (as shown by the black arrow). The 400× image is the magnified view of the area denoted by the dashed boxes in the 200× image. Scale bar: 50 μm.
