# Supplementary figures and images for: GMSC-Derived Exosomes Combined with a Chitosan/Silk Hydrogel Sponge Accelerates Wound Healing in a Diabetic Rat Skin Defect Model
Source: Front Physiol. 2017 Nov 7;8:904. doi: 10.3389/fphys.2017.00904 (PMC5681946; doi:10.3389/fphys.2017.00904)

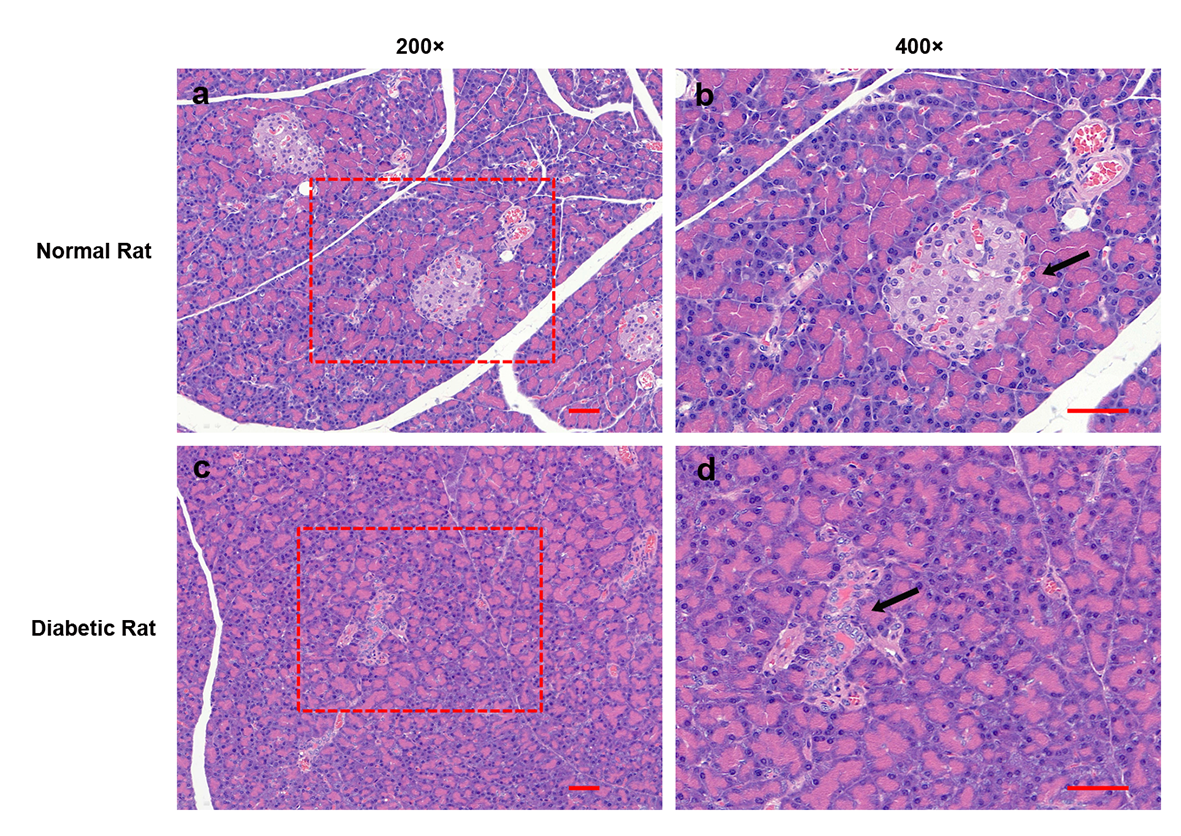

Supplement: Figure S1 — H&E staining of pancreatic tissue in normal and diabetic rats. (A,B) Normal architecture of the pancreas in the control rat. The black arrows show the islet cells. (C,D) The section of the diabetic rat pancreas displayed pathological changes. The acinar cells were swollen and the islet β-cells were damaged (as shown by the black arrow). The 400× image is the magnified view of the area denoted by the dashed boxes in the 200× image. Scale bar: 50 μm. [file Image1.TIF]
